# Visual acuity, near phoria and accommodation in myopic children using spectacle lenses with aspherical lenslets: results from a randomized clinical trial

**DOI:** 10.1186/s40662-022-00304-3

**Published:** 2022-09-01

**Authors:** Yingying Huang, Xue Li, Chu Wang, Fengchao Zhou, Adeline Yang, Hao Chen, Jinhua Bao

**Affiliations:** 1 Eye Hospital and School of Ophthalmology and Optometry, Wenzhou Medical University, 270 West Xueyuan Road, Wenzhou, 325027 Zhejiang China; 2 National Clinical Research Center for Ocular Diseases, Wenzhou, Zhejiang China; 3 Wenzhou Medical University-Essilor International Research Center (WEIRC), Wenzhou, Zhejiang China; 4 R&D AMERA, Essilor International, Singapore, Singapore

**Keywords:** Aspherical lenslets, Accommodation, Contrast visual acuity, Near phoria

## Abstract

**Objectives:**

To investigate the short- and long-term effects of myopia control spectacle lenses with highly aspherical lenslets (HAL) and slightly aspherical lenslets (SAL) on visual function and visual quality using data obtained from a randomized controlled clinical trial.

**Methods:**

This was a prospective, randomized, controlled, and double-blinded study; 170 myopic children aged 8–13 years were randomly assigned to the HAL, SAL, or single-vision spectacle lenses (SVL) groups. Distance and near visual acuity (VA) at high (100%) and low (10%) contrast in photopic and scotopic conditions, near phoria, stereoacuity, and accommodative lag, microfluctuations (AMFs), amplitude (AA) were measured after wearing lenses for 10 min, 6 months, and 12 months.

**Results:**

In total, 161 subjects completed all follow-up in 12 months and were included in the analysis. After 10 min of wearing, the HAL and SAL groups had lower scotopic and low-contrast VA than the SVL group (decreased 0.03–0.08 logMAR and 0.01–0.04 logMAR in different VAs in the HAL and SAL groups, respectively, all *P* < 0.05). The reduction in VA was recovered at 12 months as the HAL and SAL groups exhibited significant VA improvements, and the VA was not different among the three groups (all *P* > 0.05). The HAL and SAL groups had significantly larger AMFs than the SVL group (HAL vs. SAL vs. SVL: 0.21 ± 0.08 D vs. 0.16 ± 0.05 D vs. 0.15 ± 0.06 D at baseline, 0.19 ± 0.07 D vs. 0.17 ± 0.05 D vs. 0.13 ± 0.07 D at 12 months, all *P* < 0.05). There were no significant differences in accommodative lag, AA, or phoria between the groups (all *P* > 0.05). The HAL and SAL groups had reduced stereoacuity compared to the SVL group at baseline (70’ vs. 60’ vs. 50’, *P* = 0.005), but no difference was observed at 12 months (70’ vs. 70’ vs. 70’, *P* = 0.11).

**Conclusions:**

HAL and SAL have no significant influence on accommodation and phoria except had larger AMF than SVL. Scotopic VA and low-contrast VA are reduced with short-term HAL and SAL use but recovered to be at same level with the SVL after 1 year of use.

*Trial registration* Chinese Clinical Trial Registry: ChiCTR1800017683. Registered on 9 August 2018. http://www.chictr.org.cn/showproj.aspx?proj=29789

**Supplementary Information:**

The online version contains supplementary material available at 10.1186/s40662-022-00304-3.

## Introduction

Myopia has become an international public health issue in recent decades. Many methods have been used to control myopia progression. According to animal studies conducted in different species, peripheral myopic defocus has been hypothesized to be an effective way to control myopia [1–4]. Lenses based on this hypothesis, such as orthokeratology (OK) lenses [5–7], bifocal/multifocal soft contact lenses [8–10], and defocus incorporated multiple-segment (DIMS) spectacle lenses [11], have also been investigated for myopia control in children. In addition to assessing their efficacy in myopia control, visual quality and visual function after introducing peripheral defocus are essential aspects that should be fully explored.

Several previous studies have reported the effects of these myopia interventions on visual quality and visual function. After wearing OKs, accommodative parameters such as lag, amplitude, and facility have been shown to be improved [12, 13], heterophoria to become more exophoric [14, 15], and low contrast visual acuity (VA) to decrease due to an increase in high-order aberration [16]. Similar changes have been found after wearing multifocal contact lenses except for no significant improvement in accommodation [17–19]. No significant difference in changes of high- and low-contrast VAs, phoria, or accommodation have been found after 2-year wearing of DIMS lenses and single-vision spectacle lenses (SVL) [20]. Different from the honeycomb multizone design of DIMS lenses, Bao et al. [21, 22] recently introduced a novel lens design, spectacle lenses with concentric rings formed by contiguous aspherical lenslets. The 1-year results showed 0.11 mm and 0.33 D decreases in axial length (AL) elongation and spherical equivalent refraction (SER) progression in the spectacle lenses with slightly aspherical lenslets (SAL) group compared with the SVL group and 0.23 mm and 0.53 D decreases in AL and SER progression in the spectacle lenses with highly aspherical lenslets (HAL) group compared with the SVL group [21]. Two studies have shown that HAL and SAL have low impact on short-term visual acuity and contrast sensitivity in children and adult population [23, 24]. However, the long-term impacts on various aspects of visual function and visual quality of HAL and SAL in myopic children are yet to be investigated, which is the aim of this study.

## Methods

### Study design and subjects

Details of the study design have been described previously [21]. The Ethics Committee of the Eye Hospital of Wenzhou Medical University approved this prospective, randomized, controlled, and double-blind study (Y2018-054), and all work was carried out following the tenets of the Declaration of Helsinki. Written informed consent and assent were obtained from the parents and children after verbal and written explanations of the objectives and possible consequences of the study. A total of 170 Chinese children 8–13 years old with SER between − 0.75 D and − 4.75 D were included and randomized in a 1:1:1 ratio to two experimental groups (SAL or HAL group) or a control group (SVL group). Every participant was provided a new prescription and a pair of new spectacles at each visit. The measurements were always made with newly dispensed spectacles with an up-to-date full correction. HAL, SAL, and SVL all underwent the same lens processing verification and frame adjustment to achieve good fitting, and the lens center was located in front of the pupil. When the 24-month point (final visit) was reached, the participants were given a lens type of their choice (HAL or SVL) in their new spectacles, and since this might be different from that worn during the 2 years of the study, compatible 24-month visual tests could not be made. Therefore, this article only presented the 1st year’s results to compare the effects of different lenses on visual acuity and visual function. The subjects were followed at baseline (after wearing lenses for 10 min), 6 months, and 12 months, and 161 subjects attended all visits [52 (95%) subjects in the SVL group, 55 (96%) in the SAL group, and 54 (93%) in the HAL group] [21].

The design of HAL and SAL has been described previously [24]. The lens contains a 9-mm center optical zone without lenslets for distance refractive error correction and 11 concentric ring configurations with contiguous aspherical lenslets (diameter 1.1 mm). The surface of the lens without lenslets also provides distance correction. The density of lenslets is approximately 40% of the total surface area.

### Outcome measures

All measurements were performed with fully corrected, newly dispensed spectacles. Participants were not strictly required to look through the central optical zone; they wore glasses according to their daily wearing habits.

The accommodation response (AR) was measured in the right eye under bilateral viewing with an open-field autorefractor (WAM-5500, Grand Seiko Co., Ltd, Japan). The target continuously presented a single Chinese character from a short story with a height of 1.8 mm (0.31°) at the center of a laptop screen 33 cm in front of the right eye. The autorefractor was set to a high-speed mode to record refractive data at 5 Hz for 60 s continuous measurements. The accommodative stimulus and AR at the corneal plane were calculated using the equations which corrected for the effect of the spectacle lenses on autorefractor readings [25]. In terms of accommodative microfluctuation (AMF) calculation, the autorefractor readings from the first 10 s (50 numerical) and the anomalous autorefractor readings (such as less than − 6.00 D, greater than 0.00 D, or the difference of more than 1.00 D in the two consecutive values) were excluded, and the standard deviation (SD) was calculated as AMFs from the remaining data [26]. Accommodative lag was calculated by subtracting the AR from the stimulus [26].

The accommodative amplitude (AA) of the right eye was measured by a push-up method using a Royal Air Force ruler (Haag-Streit England, Essex, United Kingdom). The subjects were instructed to keep the rightmost letter of the smallest line seen of the N-series target on the ruler clear and report the first sustained blur. The researcher recorded the distance from the eye to the target when the participant reported a sustained blur. AA was defined as the reciprocal of distance and expressed in diopters. Three measurements were recorded and averaged.

High (100% contrast) and low (10% contrast) contrast VA at 5.5 m (distance) was measured using a multifunctional visual acuity test (MFVA-100, Shenzhen BriteEye Medical Tech, China) [21], and VA at 40 cm (near) was measured using the Logarithmic Contrast Acuity Chart 2000 “New ETDRS” (Chart “1” for 100% contrast, Chart “2” for 10% contrast, recorded in logMAR) at two illuminances (photopic 200 lx and scotopic 5 lx). All VA measurements were performed with binocular viewing, and monocular viewing was additionally measured for 100% contrast photopic distance and near VA.

Near horizontal phoria was measured using the modified Thorington test card at 33 cm. Three measurements were taken and averaged. Positive values of phoria denote esophoria, negative values denote exophoria, and values between − 0.3 and + 0.3 are considered orthophoria. Stereoacuity was measured using a Randot stereotest (Stereo Optical Co, Inc., U.S.A.) at 40 cm.

### Data analysis

The data are expressed as the mean (± SD) for continuous variables and the median (quartile range) for categorical variables. Repeated-measures analysis of variance (RM-ANOVA) was used to compare differences between visits, with the treatment group as the independent factor for continuous variables. Post hoc comparisons were conducted for each pair of visits and groups for significant outcomes. The nonparametric test, Kruskal–Wallis test for within visits, and Friedman test for within groups were used for categorical variables (such as stereoacuity). Statistical significance was determined at *P* values less than 0.05 that were adjusted for multiple comparisons.

## Results

The baseline characteristics of the participants in each group are shown in Table 1. The SAL group had a higher proportion of females and a shorter AL than the other two groups at baseline.

**Table 1:** Baseline characteristics of participants who completed the 12-month follow-up

	HAL	SAL	SVL	ANOVA or χ^2^ test, *P* value
Sample size	54	55	52	
Age (years)	10.65 (1.15)	10.17 (1.24)	10.37 (1.27)	0.12
Gender (M/F)	26/28	18/37	29/23	0.05
Cycloplegic SER (D)	− 2.70 (1.02)	− 2.31 (0.99)	− 2.46 (0.90)	0.12
AL (mm)	24.76 (0.68)	24.43 (0.76)	24.77 (0.65)	0.02*

*HAL* = spectacle lenses with highly aspherical lenslets; *SAL* = spectacle lenses with slightly aspherical lenslets; *SVL* = single-vision spectacle lenses; *SER* = spherical equivalent refraction; *AL* = axial length

*P < 0.05. Data are expressed as mean (SD)

The computer system for collecting AR data crashed during the 12-month visits; consequently, data from 62 subjects were lost at 12 months, and only data from 99 subjects were available. Baseline characteristics among the three groups were compared again in the analysis data group (with AR data recorded at 12 months) and the missing data group (with AR data lost recorded at 12 months; Additional file 1: Table S1). Lag and AMF data at baseline and 6 months were compared, and no significant difference was found between the missing data (62 subjects) and analysis data (99 subjects), except for the SAL group at the 6-month visit, which had a statistically significant but not clinically significant difference (Table 2). Therefore, for the analysis of lag and AMF, only 99 subjects were included.

**Table 2:** Comparisons of participants with accommodation data recorded at 12 months (analysis data) and participants with lost data recorded at 12 months (missing data) at baseline and 6 months

	Analysis data	Missing data	t-test, *P* value
Baseline			
HAL group			
N	37	17	
Lag (D)	0.87 (0.29)	0.86 (0.35)	0.91
AMF (D)	0.21 (0.08)	0.19 (0.07)	0.42
SAL group			
N	31	24	
Lag (D)	0.94 (0.26)	0.95 (0.23)	0.93
AMF (D)	0.16 (0.05)	0.15 (0.06)	0.44
SVL group			
N	31	21	
Lag (D)	0.85 (0.27)	0.89 (0.33)	0.70
AMF (D)	0.15 (0.06)	0.17 (0.06)	0.51
6 months			
HAL group			
N	37	17	
Lag (D)	0.76 (0.31)	0.79 (0.40)	0.73
AMF (D)	0.16 (0.07)	0.18 (0.05)	0.36
SAL group			
N	31	24	
Lag (D)	0.74 (0.27)	0.72 (0.26)	0.80
AMF (D)	0.15 (0.05)	0.12 (0.04)	0.02*
SVL group			
N	31	21	
Lag (D)	0.77 (0.21)	0.81 (0.21)	0.55
AMF (D)	0.11 (0.04)	0.14 (0.06)	0.08

*HAL* = spectacle lenses with highly aspherical lenslets; *SAL* = spectacle lenses with slightly aspherical lenslets; *SVL* = single-vision spectacle lenses; *AMF* = accommodative microfluctuation. *P < 0.05. Data are expressed as mean (SD)

There were no significant differences in accommodative lag between the groups at any visit. The SAL group showed a significant decrease in lag with time, while the other two groups did not change over time (Table 3). There were significant differences in AMFs between the groups at each visit (all *P* < 0.01, Table 3), and AMFs were greater in the HAL and SAL groups than in the SVL group. The HAL and SVL groups showed a small but significant difference in AMF over time (*P* < 0.05), and no difference was found in the SAL group. No significant differences in AA were found between the groups at each visit (*P* > 0.05), but a significant increase of approximately 1.00 D was found in the SAL and HAL groups after 1 year (*P* < 0.05); the SVL group did not show a change in AA (*P* = 0.24). Comparisons by RM-ANOVA with data adjusted for baseline AL and sex showed same significance (Additional file 1: Table S2).

**Table 3:** Comparison of accommodative lag, microfluctuation (AMF), and amplitude in participants with accommodation data successfully recorded at all three visits

	HAL	SAL	SVL	RM-ANOVA, *P* value		
				Time	Group	Time × Group
Lag (D)						
N	37	31	31			
Baseline	0.87 (0.29)	0.94 (0.26)	0.85 (0.27)	< 0.001*	0.97	0.44
6 months	0.76 (0.31)	0.74 (0.27)	0.77 (0.21)			
12 months	0.77 (0.26)	0.77 (0.21)	0.80 (0.17)			
AMF (D)						
N	37	31	31			
Baseline	0.21 (0.08)	0.16 (0.05)	0.15 (0.06)	0.001*	< 0.001*	0.40
6 months	0.16 (0.07)	0.15 (0.05)	0.11 (0.04)			
12 months	0.19 (0.07)	0.17 (0.05)	0.13 (0.07)			
Amplitude (D)						
N	54	55	52			
Baseline	10.53 (2.90)	11.35 (3.22)	11.31 (3.54)	< 0.001*	0.16	0.96
6 months	11.21 (2.54)	11.83 (2.46)	11.71 (2.95)			
12 months	11.57 (2.16)	12.43 (2.16)	12.05 (2.75)			

*HAL* = spectacle lenses with highly aspherical lenslets; *SAL* = spectacle lenses with slightly aspherical lenslets; *SVL* = single-vision spectacle lenses. *P < 0.05. Data are expressed as mean (SD)

**Fig. 1:**
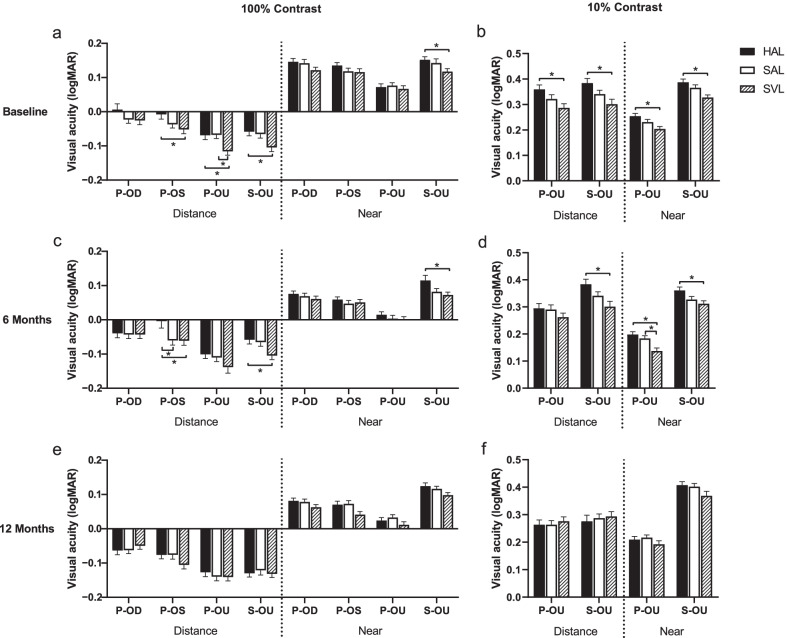
Visual acuity in 100% and 10% contrast, photopic (200 lx) and scotopic (5 lx), distance (5.5 m) and near (40 cm) at baseline (**a** and **b**), 6 months (**c** and **d**) and 12 months (**e** and **f**). Error bars represent one standard error of the mean. HAL, spectacle lenses with highly aspherical lenslets; SAL, spectacle lenses with slightly aspherical lenslets; SVL, single-vision spectacle lenses; P, photopic; S, scotopic. *P < 0.05

At baseline, after wearing lenses for 10 min, the participants in the SVL group showed slightly higher VA than the other two groups in high-contrast distance VA and in all low-contrast VA (Fig. 1). However, differences in VA between the groups were all less than one line (0.1 logMAR). Comparison between visits showed significant improvements in all VA measurements in the SAL and HAL groups and for only near VA in the SVL group (all *P* < 0.05). At the 12-month visit, there was no significant difference in any VA measurement between the groups. Comparisons with data adjusted for baseline AL and sex showed same significance (Additional file 1: Table S2).

Comparison of near phoria showed no differences between the three groups at each visit (Table 4) and showed no significant difference over time. Stereoacuity in the HAL group was worse than that in the SVL and SAL groups at the baseline and 6-month visits, while no significant difference was found at 12 months (Table 5).

**Table 4:** Comparison of near phoria (Δ) among the three groups

	HAL	SAL	SVL	RM-ANOVA, *P* value		
				Time	Group	Time × Group
Baseline	− 1.86 (6.76)	− 2.24 (6.60)	− 2.37 (6.52)	0.07	0.67	0.92
6 months	− 1.79 (6.09)	− 2.88 (6.15)	− 3.47 (5.14)			
12 months	− 2.96 (5.00)	− 2.48 (6.95)	− 3.62 (5.53)			

*HAL* = spectacle lenses with highly aspherical lenslets; *SAL* = spectacle lenses with slightly aspherical lenslets; *SVL* = single-vision spectacle lenses. Data are expressed as mean (SD)

**Table 5:** Comparison of stereoacuity(s) among the three groups

	HAL	SAL	SVL	Kruskal-Wallis test, *P* value
Baseline	70 (30, 70)	60 (40, 70)	50 (40, 70)	0.005*
6 months	70 (52.5, 70)	70 (30, 70)	60 (40, 70)	0.01*
12 months	70 (50, 70)	70 (30, 70)	70 (30, 70)	0.11
Friedman test, *P* value	0.59	0.69	0.53	

*HAL* = spectacle lenses with highly aspherical lenslets; *SAL* = spectacle lenses with slightly aspherical lenslets; *SVL* = single-vision spectacle lenses. *P < 0.05. Data are expressed as median (quartile range)

## Discussion

This study aimed to determine the influence of spectacle lenses with aspherical lenslets on visual function and visual quality after 1 year of use. The results showed that the two different aspherical lenslet designs had no clinically meaningful influence on near phoria, accommodative lag, or amplitude. SAL and HAL exhibited a slightly lower performance than SVL in scotopic and low-contrast VA at the initial use, but after 12 months, VA was recovered to be comparable to SVL in all conditions. Comparisons of stereoacuity were consistent with those for VA. Only in AMFs was there a difference between the HAL and the control groups at baseline and after 1 year.

Unlike bifocals and progressive addition lenses, lenses with aspherical lenslets do not decrease the accommodative lag compared with SVL. The additions of bifocals and progressive addition lenses are continuous and can form a clear image on the retina, and near additions are supposed to decrease the accommodative demand and thus reduce the accommodative lag during near-vision work [27–29]. However, aspherical lenslets are discontinuous and cannot form a clear image, so they are less likely to significantly affect the lag [30]. However, in this study, the lag in the three groups showed a certain decrease. Presumably, some subjects were undercorrected more than − 0.50D or uncorrected before being included in the study (79.6%, 78.2%, and 84.6% in the HAL, SAL, and SVL, respectively); therefore, when they were given the full-correction spectacles and were asked to wear them all the time, including during near work, their accommodation improved.

The HAL and SAL groups were found to have larger AMFs than the SVL group. There are two possible explanations for this. One is that the aberrations conferred by aspherical lenslets influence accommodation, but the lag did not differ between groups. Another is that the larger AMFs may be due to the measurement partly through the lenslet zone, causing the variational accommodation demand from visual signals coexisting with and without aspherical lenslets.

AA was measured by a push-up method in this study, and the results were slightly lower than the normal range at the subjects’ ages. Previous studies have shown that AA decreases significantly with time in children at the primary school age [20, 31–33]. In this study, after wearing spectacles for 1 year, subjects in the SAL and HAL groups showed significant improvements in AA of more than 1.00 D; the AA in the SVL group subjects also increased by approximately 0.74 D (*P* = 0.24), and there was no difference between the groups. Similar to the decrease in lag, full correction can improve AA compared with undercorrection. Another possible reason is learning bias (i.e., familiarity with the measurements) at the 6-month and 12-month visits.

Several researchers have investigated the effects of myopia control spectacle lenses on vision. Lam et al. found that subjects in SVL and DIMS groups had no difference in VA. But subjects in their study were tested VA with SVL in both SVL and DIMS groups [20]. They aimed to figure out whether the DIMS lenses had any effect on the children’s vision and visual function, rather than the immediate effects of the lenses on vision and visual function. In this study, we tested the effects of wearing HAL and SAL lenses on children’s vision and visual function in daily life, participants were tested with HAL and SAL. In our study, visual acuity was measured in different conditions to include as many conditions encountered in the real world, such as photopic and scotopic lighting, distance and near, high and low contrast. At baseline, wearing lenses for 10 min resulted in lower performance in the HAL group. The differences were 0.01 to 0.05 logMAR between the SAL and SVL groups and 0.03 to 0.08 logMAR between the HAL and SVL groups in different VA, which was statistically significant but had less clinical meaning and was not considered to have an influence on daily life. After the lenses were used for 6 months, all VA in the SAL and HAL groups showed significant improvements, but SAL and HAL still have significant impact on VA in the scotopic and low-contrast VA. After 12 months of wearing, there was no difference between the experimental groups and the SVL group in any VA. During the VA tests, the participants were asked to wear spectacles in their accustomed ways, and they were not strictly asked to look through the central optical zone. When the subjects first used the HAL and SAL at baseline, it could be that the participants did not fixate completely through the center of the lens (without lenslets area) but partly through the central area and partly through the peripheral aspherical lenslets area. A previous study found that low-contrast scotopic VA was reduced when fixating through the lenslets area of HAL and SAL [23]. Therefore, during the 1-year wearing period, children learned to use the lenses (find the clear center area), the influence of aspherical lenslets was reduced, and the VA improved significantly. Another possibility is blur adaptation [34–37]. A previous study showed that blur adaptation to 1.00 D at the fovea for 30 min can improve vision by approximately 0.07 logMAR [34]. That is, although HAL and SAL would introduce peripheral blurring while wearing, subjects adapted to blurring with 6 and 12 months of wearing, resulting in improvements in vision.

The magnitude and direction of phoria were not significantly different between the groups or over time. Participants included in this study were all children with phoria, children with intermittent exotropia could be included to explore the influence of aspherical lenslets on their phoria and binocular vision in the following study.

The stereoacuity results showed a similar trend to the VA results. Reduced stereoacuity was found in the HAL group compared to the SVL group at baseline and 6-month visits, but the differences were small and had no clinical significance. In general, the participants in this study had lower stereoacuity than those in other studies [38, 39], and more than 50% of the subjects in this study had a low stereoacuity (equal to or more than 70″). The causes need to be explored further, but a more precise method for assessing stereoacuity will be helpful for understanding possible influences.

One limitation in this study is that subjects in the experimental groups did not perform the measurements with SVL at baseline to exclude individual differences, but the randomized design of this study would avoid the difference substantially. Previous studies have found that pupil diameter is an important factor in the myopia control effect of OK [40, 41], so pupil diameter may also affect the myopia control efficacy of HAL and SAL. Pupil diameter is affected by accommodation and environmental factors. Not measuring pupil diameter is a limitation in this study. Another limitation is the missing data at 12 months, but the missing data were considered to not affect the results, as there was no significant difference between the analysis data and missing data.

## Conclusions

In conclusion, spectacle lenses with aspherical lenslets have no significant influence on accommodation and near phoria except resulting in larger AMFs than SVL. Children who wear lenses with lenslets need a longer period to develop the same clear vision as those wearing SVL in scotopic and low contrast, especially with HAL. After wearing lenses with lenslets for 1 year, participants experienced the same VA as participants who wore SVL. This study showed that highly aspherical lenslets influenced scotopic and low-contrast VA and AMFs more; however, highly aspherical lenslets have a better effect on slowing myopia progression. In future research, subjects with abnormal visual function should be included to observe the influence of the lenslets to provide clinical guidance.

## Supplementary Information

**Additional file 1: Table S1. **Baseline characteristics of missing data subjects and complete data subjects. **Table S2.** Comparisons over time and among groups by repeated-measures ANOVA (RM-ANOVA) with adjusted for baseline axial length (AL) and sex.

## Data Availability

The datasets used and analyzed for the present study are available from the corresponding authors upon reasonable request.

## Abbreviations

HAL: Spectacle lenses with highly aspherical lenslets
SAL: Spectacle lenses with slightly aspherical lenslets
SVL: Single-vision spectacle lenses
VA: Visual acuity
AMF: Accommodative microfluctuation
AA: Accommodative amplitude
OK: Orthokeratology
DIMS: Defocus incorporated multiple-segment
AL: Axial length
SER: Spherical equivalent refraction
AR: Accommodation response
D: Diopter
