# Supplementary material for: Visual acuity, near phoria and accommodation in myopic children using spectacle lenses with aspherical lenslets: results from a randomized clinical trial
Source: Eye Vis (Lond). 2022 Sep 1;9:33. doi: 10.1186/s40662-022-00304-3 (PMC9434851; doi:10.1186/s40662-022-00304-3)
Supplement: Supplementary file 1 — Additional file 1: Table S1. Baseline characteristics of missing data subjects and complete data subjects. Table S2. Comparisons over time and among groups by repeated-measures ANOVA (RM-ANOVA) with adjusted for baseline axial length (AL) and sex. [file 40662_2022_304_MOESM1_ESM.docx]

Table S1. Baseline characteristics of missing data subjects and complete data subjects

|  | HAL | SAL | SVL | One way ANOVA, *P* value |
| --- | --- | --- | --- | --- |
| Analysis data subjects |  |  |  |  |
| Sample size | 37 | 31 | 31 |  |
| Age | 10.59 (1.12) | 9.81 (1.22) | 10.52 (1.12) | 0.01* |
| Gender (M/F) | 19/18 | 11/20 | 17/14 | 0.26 |
| Cycloplegic SER (D) | −2.65 (1.00) | −2.33 (1.02) | −2.44 (0.84) | 0.37 |
| AL (mm) | 24.70 (0.68) | 24.40 (0.73) | 24.80 (0.66) | 0.06 |
| Missing data subjects |  |  |  |  |
| Sample size | 17 | 24 | 21 |  |
| Age | 10.76 (1.25) | 10.54 (1.22) | 10.14 (1.46) | 0.33 |
| Gender (M/F) | 7/10 | 7/17 | 12/9 | 0.17 |
| Cycloplegic SER (D) | −2.79 (1.08) | −2.29 (0.97) | −2.49 (1.00) | 0.30 |
| AL (mm) | 24.87 (0.69) | 24.47 (0.81) | 24.73 (0.66) | 0.20 |
| HAL = spectacle lenses with highly aspherical lenslets; SAL = spectacle lenses with slightly aspherical lenslets; SVL = single-vision spectacle lenses; SER = spherical equivalent refraction; AL = axial length. **P* < 0.05. Data are expressed as mean (SD). | | | | |

Table S2. Comparisons over time and among groups by repeated-measures ANOVA (RM-ANOVA) with adjusted for baseline axial length (AL) and sex

|  |  |  | adjusted for baseline AL and sex | | | RM-ANOVA, *P* value | | |
| --- | --- | --- | --- | --- | --- | --- | --- | --- |
|  |  |  | HAL | SAL | SVL | Time | Group | Time*Group |
| Visual acuity (logMAR) | 100%, far, photopic in OD | Baseline | 0.01 (0.10) | −0.02 (0.10) | −0.03 (0.10) | 0.24 | 0.79 | 0.31 |
|  |  | 6 Months | −0.04 (0.09) | −0.04 (0.10) | −0.04 (0.09) |  |  |  |
|  |  | 12 Months | −0.07 (0.08) | −0.06 (0.08) | −0.05 (0.08) |  |  |  |
|  | 100%, far, photopic in OS | Baseline | −0.01 (0.09) | −0.03 (0.10) | −0.05 (0.09) | 0.02* | 0.003* | 0.20 |
|  |  | 6 Months | −0.00 (0.12) | −0.06 (0.12) | −0.06 (0.12) |  |  |  |
|  |  | 12 Months | −0.08 (0.09) | −0.07 (0.09) | −0.11 (0.09) |  |  |  |
|  | 100%, far, photopic in OU | Baseline | −0.07 (0.09) | −0.07 (0.09) | −0.12 (0.09) | 0.05 | 0.02* | 0.38 |
|  |  | 6 Months | −0.10 (0.10) | −0.11 (0.10) | −0.14 (0.11) |  |  |  |
|  |  | 12 Months | −0.13 (0.09) | −0.14 (0.09) | −0.14 (0.09) |  |  |  |
|  | 100%, far, scotopic in OU | Baseline | −0.06 (0.09) | −0.07 (0.09) | −0.11 (0.09) | 0.03* | 0.02* | 0.14 |
|  |  | 6 Months | −0.09 (0.09) | −0.11 (0.09) | −0.14 (0.09) |  |  |  |
|  |  | 12 Months | −0.13 (0.09) | −0.12 (0.09) | −0.13 (0.09) |  |  |  |
|  | 100%, near, photopic in OD | Baseline | 0.15 (0.07) | 0.14 (0.07) | 0.12 (0.07) | 0.49 | 0.07 | 0.95 |
|  |  | 6 Months | 0.08 (0.07) | 0.07 (0.07) | 0.06 (0.06) |  |  |  |
|  |  | 12 Months | 0.08 (0.06) | 0.08 (0.06) | 0.06 (0.06) |  |  |  |
|  | 100%, near, photopic in OS | Baseline | 0.14 (0.07) | 0.12 (0.07) | 0.12 (0.07) | 0.41 | 0.13 | 0.17 |
|  |  | 6 Months | 0.06 (0.07) | 0.05 (0.07) | 0.05 (0.06) |  |  |  |
|  |  | 12 Months | 0.07 (0.07) | 0.08 (0.07) | 0.04 (0.07) |  |  |  |
|  | 100%, near, photopic in OU | Baseline | 0.07 (0.07) | 0.07 (0.07) | 0.07 (0.06) | 0.07 | 0.30 | 0.83 |
|  |  | 6 Months | 0.01 (0.07) | 0.01 (0.07) | 0.00 (0.06) |  |  |  |
|  |  | 12 Months | 0.02 (0.07) | 0.03 (0.07) | 0.01 (0.06) |  |  |  |
|  |  |  |  |  |  |  |  |  |
|  |  |  |  |  |  |  |  |  |
|  |  |  | adjusted for baseline AL and sex | | | RM-ANOVA, *P* value | | |
|  |  |  | HAL | SAL | SVL | Time | Group | Time*Group |
| Visual acuity (logMAR) | 100%, near, scotopic in OU | Baseline | 0.15 (0.07) | 0.15 (0.07) | 0.12 (0.07) | 0.89 | 0.001* | 0.52 |
|  |  | 6 Months | 0.12 (0.09) | 0.08 (0.09) | 0.07 (0.09) |  |  |  |
|  |  | 12 Months | 0.12 (0.07) | 0.12 (0.07) | 0.10 (0.06) |  |  |  |
|  | 10%, far, photopic in OU | Baseline | 0.36 (0.12) | 0.32 (0.13) | 0.29 (0.13) | 0.77 | 0.22 | 0.07 |
|  |  | 6 Months | 0.30 (0.12) | 0.29 (0.13) | 0.26 (0.12) |  |  |  |
|  |  | 12 Months | 0.26 (0.12) | 0.26 (0.12) | 0.28 (0.12) |  |  |  |
|  | 10%, far, scotopic in OU | Baseline | 0.38 (0.13) | 0.34 (0.13) | 0.30 (0.13) | 0.75 | 0.15 | 0.03 |
|  |  | 6 Months | 0.31 (0.13) | 0.30 (0.13) | 0.26 (0.14) |  |  |  |
|  |  | 12 Months | 0.28 (0.14) | 0.28 (0.14) | 0.30 (0.14) |  |  |  |
|  | 10%, near, photopic in OU | Baseline | 0.26 (0.07) | 0.23 (0.07) | 0.20 (0.08) | 0.23 | <0.001* | 0.21 |
|  |  | 6 Months | 0.20 (0.08) | 0.18 (0.08) | 0.14 (0.08) |  |  |  |
|  |  | 12 Months | 0.21 (0.08) | 0.22 (0.09) | 0.19 (0.09) |  |  |  |
|  | 10%, near, scotopic in OU | Baseline | 0.39 (0.09) | 0.37 (0.09) | 0.33 (0.09) | 0.79 | <0.001* | 0.63 |
|  |  | 6 Months | 0.36 (0.09) | 0.33 (0.09) | 0.31 (0.09) |  |  |  |
|  |  | 12 Months | 0.41 (0.10) | 0.40 (0.10) | 0.37 (0.10) |  |  |  |
| Accommodation (D) | Lag | Baseline | 0.88 (0.33) | 0.94 (0.38) | 0.86 (0.36) | 0.50 | 0.98 | 0.48 |
|  |  | 6 Months | 0.76 (0.32) | 0.74 (0.36) | 0.77 (0.34) |  |  |  |
|  |  | 12 Months | 0.77 (0.04) | 0.76 (0.30) | 0.80 (0.29) |  |  |  |
|  | Microfluctuation | Baseline | 0.21 (0.08) | 0.16 (0.09) | 0.16 (0.09) | 0.28 | <0.001* | 0.24 |
|  |  | 6 Months | 0.16 (0.06) | 0.16 (0.07) | 0.12 (0.06) |  |  |  |
|  |  | 12 Months | 0.19 (0.08) | 0.17 (0.09) | 0.13 (0.09) |  |  |  |
|  | Amplitude | Baseline | 10.58 (3.25) | 11.26 (3.29) | 11.35 (3.29) | 0.40 | 0.16 | 0.91 |
|  |  | 6 Months | 11.18 (2.67) | 11.92 (2.71) | 11.64 (2.70) |  |  |  |
|  |  | 12 Months | 11.57 (2.36) | 12.48 (2.40) | 12.00 (2.39) |  |  |  |
|  |  |  | adjusted for baseline AL and sex | | | RM-ANOVA, *P* value | | |
|  |  |  | HAL | SAL | SVL | Time | Group | Time*Group |
| Phoria at 33 cm (Δ) |  | Baseline | −1.86 (6.63) | −1.79 (6.73) | −2.90 (6.66) | 0.48 | 0.65 | 0.96 |
|  |  | 6 Months | −2.79 (5.74) | −2.76 (5.83) | −3.28 (5.77) |  |  |  |
|  |  | 12 Months | −2.59 (5.94) | −2.49 (6.03) | −3.56 (5.96) |  |  |  |
| HAL = spectacle lenses with highly aspherical lenslets; SAL = spectacle lenses with slightly aspherical lenslets; SVL = single-vision spectacle lenses; 100%, 100% contrast; 10%, 10% contrast. **P* < 0.05. Data are expressed as mean (SD). | | | | | | | | |
